# *HLA-G* 3’UTR Polymorphisms Impact the Prognosis of Stage II-III CRC Patients in Fluoropyrimidine-Based Treatment

**DOI:** 10.1371/journal.pone.0144000

**Published:** 2015-12-03

**Authors:** Marica Garziera, Ettore Bidoli, Erika Cecchin, Enrico Mini, Stefania Nobili, Sara Lonardi, Angela Buonadonna, Domenico Errante, Nicoletta Pella, Mario D’Andrea, Francesco De Marchi, Antonino De Paoli, Chiara Zanusso, Elena De Mattia, Renato Tassi, Giuseppe Toffoli

**Affiliations:** 1 Experimental and Clinical Pharmacology Unit, Centro di Riferimento Oncologico (CRO), IRCCS, Aviano National Cancer Institute, via F. Gallini 2, 33081 Aviano, Italy; 2 Epidemiology and Biostatistic Unit, Centro di Riferimento Oncologico (CRO), IRCCS, Aviano National Cancer Institute, via F. Gallini 2, 33081 Aviano, Italy; 3 Section of Internal Medicine, Department of Experimental and Clinical Medicine, University of Florence, Florence, Italy; 4 Section of Clinical Pharmacology and Oncology, Department of Health Sciences, University of Florence, Florence, Italy; 5 Medical Oncology Unit 1, Istituto Oncologico Veneto-IRCCS, Padua, Italy; 6 Division of Medical Oncology B; Centro di Riferimento Oncologico (CRO), IRCCS, Aviano National Cancer Institute, via F. Gallini 2, 33081 Aviano, Italy; 7 Medical Oncology Unit, Ospedale Civile di Vittorio Veneto, Vittorio Veneto (TV), Italy; 8 Medical Oncology Unit, University Hospital, Piazza S. Maria Della Misericordia, Udine, Italy; 9 Medical Oncology Unit, “San Filippo Neri Hospital”, Piazza Di S. Maria Della Pietà, Rome, Italy; 10 Surgical Oncology Department, Centro di Riferimento Oncologico (CRO), IRCCS, Aviano National Cancer Institute, via F. Gallini 2, 33081 Aviano, Italy; 11 Radiotherapy Department, Centro di Riferimento Oncologico (CRO), IRCCS, Aviano National Cancer Institute, via F. Gallini 2, 33081 Aviano, Italy; School of Medicine, Fu Jen Catholic University, TAIWAN

## Abstract

An important hallmark of CRC is the evasion of immune surveillance. HLA-G is a negative regulator of host’s immune response. Overexpression of HLA-G protein in primary tumour CRC tissues has already been associated to worse prognosis; however a definition of the role of immunogenetic host background is still lacking. Germline polymorphisms in the 3’UTR region of *HLA-G* influence the magnitude of the protein by modulating HLA-G mRNA stability. Soluble HLA-G has been associated to 3’UTR +2960 Ins/Ins and +3035 C/T (lower levels) and +3187 G/G (high levels) genotypes. *HLA-G* 3’UTR SNPs have never been explored in CRC outcome. The purpose of this study was to investigate if common *HLA-G* 3’UTR polymorphisms have an impact on DFS and OS of 253 stage II-III CRC patients, after primary surgery and ADJ-CT based on FL. The 3’UTR was sequenced and SNPs were analyzed for their association with survival by Kaplan-Meier and multivariate Cox models; results underwent internal validation using a resampling method (bootstrap analysis). In a multivariate analysis, we estimated an association with improved DFS in *Ins* allele (Ins/Del +Ins/Ins) carriers (HR 0.60, 95% CI 0.38–0.93, *P* = 0.023) and in patients with +3035 C/T genotype (HR 0.51, 95% CI 0.26–0.99, *P* = 0.045). The +3187 G/G mutated carriers (G/G *vs* A/A+A/G) were associated to a worst prognosis in both DFS (HR 2.46, 95% CI 1.19–5.05, *P* = 0.015) and OS (HR 2.71, 95% CI 1.16–6.63, *P* = 0.022). Our study shows a prognostic and independent role of 3 *HLA-G* 3’UTR SNPs, +2960 14-bp INDEL, +3035 C>T, and +3187 A>G.

## Introduction

Colorectal cancer (CRC) is still a clinical burden being the third most common cancer in the United States [1] and the second leading cause of cancer death in Europe, in both women and men [2]. Recent advances in protein- and genomic-based technologies, validated predictive and prognostic biomarkers, have demonstrated that CRC should be considered as a heterogeneous disease [3–5]. Adjuvant chemotherapy (ADJ-CT) based on fluoropyrimidine (FL) is generally administered in stage II-III patients after surgical resection of the primary tumour. Despite optimal surgery and adjuvant therapies, the risk of recurrence for stages II or III disease is about 40% [6] and ~ 80% of stage II CRC patients will be disease-free even without ADJ-CT [7]. Recently, gene expression profile signatures of immune-system related genes and presence of the immune infiltrates in tumour microenvironment were shown to have an independent prognostic significance in CRC compared to classical clinical factors [8–11].

Tumour immunogenicity, cancer cell capability to escape from the host’s immune system surveillance, and immunogenetic background of the patient, represent a future challenge of current research [12]. Two main mechanisms contribute to the cancer immunoediting process [13] leading to poorly immunogenic tumour cell variants invisible to the immune system. The first is the minimization of the level of tumour associated antigens (TAA) presentation through the downregulation or loss of the human leukocyte antigen (HLA) class I expression by tumour cells [14]. The second is the competence of cancer cells in regulating the expression of the non-classical HLA class I molecules such as HLA-G [15]. The *HLA-G* gene codifies for a tolerogenic molecule with well recognized immune-inhibitory properties on both innate and adaptive immune responses [16–18]. HLA-G is highly expressed in physiological conditions in trophoblast at fetal-maternal interface and has a restricted distribution in normal tissues [19]; however, an increased expression can be induced in pathological conditions such as cancer [20,21]. Several genetic variations involved in *HLA-G* regulation have been so far described in the 5’ upstream regulatory (or promoter) region (5’URR) as well as in the 3’ untranslated region (3’UTR), while in contrast to the classical *HLA* class I loci, a lower variability in the coding regions is observed [22–24]. Increased soluble HLA-G levels in biological fluids are associated with down-modulation of the immune response in the host [15]. The *HLA-G* 3’UTR is the most studied segment of the gene due to the presence of multiple regulatory elements implicated in the modulation of HLA-G expression.

Nine (+2960 14-base pair (bp) INDEL, +3003 T>C, +3010 C>G, +3027 C>A, +3035 C>T, +3142 G>C, +3187 A>G, +3196 C>G and +3227 G>A) single nucleotide polymorphisms (SNPs) are known in this region, which can potentially alter the set of microRNAs (miRNAs) capable of binding the 3’UTR, thus influencing *HLA-G* RNA turnover, stability and splicing [25,26]. At least three of these genetic variants have been associated to the transcriptional and post transcriptional control of *HLA-G* regulation [27,28]. In particular, the presence (Ins) or absence (Del) of a 14-bp fragment (5’-ATTTGTTCATGCCT-3’) in position+2960 (14-bp INDEL, 14-bp Ins/Del) influences transcripts stability and is the most studied SNP. Presence of 14-bp *Ins* allele produces a more unstable *HLA-G* mRNA causing lower levels of the protein [25]. The G nucleotide in position +3142 favours the targeting of three miRNAs (miR-148a, -148b, and -152) leading to an increase in mRNA degradation [22]. Four-bp upstream to +3187 A>G and 9-bp downstream to +3196 C>G SNPs, two AU-rich motifs are present. The wild type +3187*A* allele is associated to decreased mRNA stability modifying an AU-rich motif [29]. Significant differences in soluble HLA-G protein levels have been observed in carriers for +2960 Ins/Ins and +3035 C/T (lower levels) [29–32], and in +3027 C/C, +3142 G/C, +3187 A/G (higher levels) [29,33] genotypes. Some 3’UTR SNPs, 14-bp INDEL in particular, have been related to the susceptibility to certain diseases such as autoimmune diseases [33], preeclampsia, transplantation, chronic inflammatory diseases and several types of cancer [30,31,34]. Notably, a recent study in human cancer (chronic lymphocytic leukemia) has reported an association with the +2960 14-bp INDEL polymorphism and plasmatic HLA-G protein levels and survival [31].

HLA-G is overexpressed in primary CRC lesions [35–37], and higher levels of the soluble protein have been detected in plasma of CRC patients compared to that of patients with benign diseases or healthy donors [38,39]. Expression of HLA-G in tumour tissues has been associated with the clinical outcome of CRC as an independent and unfavourable prognostic factor of reduced OS [40,41].

To date, the characterization of *HLA-G* genotypes, alleles and haplotypes in CRC patients has not been explored as well as their role in the prediction of CRC prognosis [42].

Considering the relevance of the 3’UTR region in the control and regulation of the HLA-G transcripts and the lack of data in literature, our purpose was searching for associations between *HLA-*G 3’UTR polymorphisms detected at the germinal level, and the disease free survival (DFS) and OS of stage II-III CRC patients in adjuvant regimen. We analyzed a cohort of 253 CRC patients to investigate if functional SNPs in the 3’UTR of *HLA-G* gene, alone or in combination in the 3’UTR haplotypes, are associated to advantage or disadvantage for DFS and OS.

## Materials and Methods

### Ethics Statement

A written Informed Consent was obtained before surgery from all the participants to the use of their blood samples and clinical data for research purpose. The study was approved by the ethical committees of the participating institutes, the Centro di Riferimento Oncologico (CRO)-Aviano National Cancer Institute, Aviano, Italy, University Hospital, Florence, Italy, Istituto Oncologico Veneto, Padua, Italy, Ospedale Civile di Vittorio Veneto, Vittorio Veneto, Italy, University Hospital “S. Maria Della Misericordia”, Udine, Italy, and “San Filippo Neri” Hospital, Rome, Italy.

### Patients and treatment

A total of 253 CRC patients with newly diagnosed, untreated, histopathologically confirmed CRC, were included from an existing prospective collection of only blood samples stored at the Experimental and Clinical Pharmacology Unit of Centro di Riferimento Oncologico (CRO)-Aviano, based on previous multicenter pharmacogenomic studies [43,44]. Eligible criteria were: stage II-III CRC, radiologically-confirmed absence of distant metastasis, age >18 years, performance status (WHO) 0–2, normal bone marrow, renal and liver function, and Caucasian ethnicity. Overall patients after diagnosis underwent primary surgery and received ADJ-CT based on fluoropyrimidine (FL) (i.e., 5-fluorouracil/folinic acid or capecitabine) [44], or FL plus oxaliplatin (FL+OXA) [43].

ADJ-CT was continued until completion of the planned cycles, recurrence, toxicity or patient refusal. Patients follow-up was measured from the time of surgery to the last contact or disease recurrence. Biological tests, pulmonary X-ray, positron emission tomography (PET) and/or computed tomography (CT) imaging alternatively with abdominal ultra-sonography were carried out every 3 months during the first 3 years after surgery. In the next 2 years PET/CT were performed every 6 months and then annually. Overall evaluations were conducted independently by the type of ADJ-CT. Recurrence was defined based on PET/CT scans in the case of metastasis presence, with pathologic confirmation made by the oncologist when necessary.

### *HLA-G* 3’UTR genotyping

A peripheral blood sample was collected in acid citrate dextrose (ACD) tubes from nearly all CRC patients. Genomic DNA was extracted from whole blood or from normal colon mucosa tissue by using the EZ1 DNA Blood or Tissue kit and the BioRobot EZ1 Workstation (QIAGEN Inc., Valencia, CA, USA). The 3’UTR of the *HLA-G* gene was amplified by polymerase chain reaction (PCR) using the already published [23] primers HLAG8F: 5’- TGTGAAACAGCTGCCCTGTGT-3’ and HLAG8R: 5’- GTCTTCCATTTATTTTGTCTCT-3’. PCR reactions were carried out in a final volume of 30 μl containing 1.25 mM MgCl2, 0.25 mM of each dNTPs, 5 pmol of each primer, about 50–200 ng of genomic DNA template, 1X PCR Buffer and 0.5 units of AmpliTaq Gold DNA polymerase (Applied Biosystems, Foster City, CA, USA). The PCR cycles were as follows: 5 mins. of initial denaturation at 94°C, 30 cycles of 45 secs. at 95°C, 45 secs. at 56°C, 60 secs. at 72°C, and the final extension step at 72°C for 7 mins. Five microliters of PCR products (344 bp in presence of deletion and 358 bp for insertion) were first analyzed by electrophoresis on 4% agarose gel stained with ethidium bromide. The remaining 25 μl of PCR reactions were purified using Diffinity RapidTip 2 tips (Sigma-Aldrich, St. Louis, MO, USA). Purified reactions (1–2 μl) were sequenced (Sanger method) by the use of the Big Dye Terminator kit (Applied Biosystems, Foster City, CA, USA) and an ABI PRISM capillary sequencer with the reverse HLAG8R primer to prevent sequence overlaps in heterozygous 14-bp samples [23]. Chromatograms were visualized with Chromas software version 2.01 and all single nucleotide polymorphisms (SNPs), and single nucleotide variants (SNVs) detected were recorded for each study participant.

### Statistical analysis

The aim of this study was to assess associations between *HLA-G* polymorphisms in the 3’UTR regulatory region and DFS and OS respectively of stage II-III CRC patients treated with ADJ-CT after primary surgery. DFS was defined as the time from date of surgery to date of clinically detectable recurrence (local, regional or distant), death from any cause, or last follow-up evaluation. OS was defined as the time from date of surgery to date of death from any cause, or last follow-up time. Longitudinal analyses were determined by means of Kaplan-Meier method (log-rank test) and Cox models. Cox proportional hazard models were used to estimate adjusted hazard ratios (HRs) and corresponding 95% confidence intervals (CIs). Associations were firstly evaluated by means of univariate models and only those that resulted statistically significant (two-sided *P*≤0.05) were included in multivariate models. Adjustment for age (continuous variable), sex (male *vs* female), stadium (II *vs* III); first tumour location (colon *vs* rectum) and type of ADJ-CT (FL-alone *vs* FL+OXA) were computed. After performing a Cox regression using the common genomic model, the associations of SNPs with clinical outcomes were also evaluated for genomic models of transmission (dominant and recessive). In a dominant model for a SNP with a major allele “A” and a minor allele “b”, the collective genotypes (“Ab”+”bb”) are compared to a reference genotype “AA”. For a recessive model, “bb” is compared to a collective (“AA”+”Ab”) reference group. The HR of a reference genotype group is arbitrarily fixed at 1.00. Survival analyses were not computed when a genotype or a haplotype was detected in only one patient. Only haplotypes with frequency >1% were included in the survival analyses. An internal validation of the study results was carried out by a bootstrap resampling technique. We ran 1000 bootstrapped Cox models adjusted for the aforementioned variables. SAS software, version 9.2 (SAS Institute Inc., Cary, NC, 1999–2001) was adopted for the estimations. Adherences of genotypic proportions to expectations under Hardy-Weinberg (HW) equilibrium and two-locus linkage disequilibrium (LD) were evaluated by means of the Haploview program v4.2. The most probable haplotype of each sample at the unknown gametic phase, was reconstructed by the use of the PHASE method (program v2.1.1) [45].

## Results

### Patient characteristics and survival analysis

The main demographic and clinical characteristics of CRC patients (N = 253) together with log-rank tests are summarized in Table 1.

**Table 1:** Clinical and demographic characteristics of patients.

Variables	N (%)	5-years	Log-rank	5-years	Log-rank
		DFS %	*P*-value^1^	OS %	*P*-value^1^
**Age, median**					
< 62.5 years	122 (48)	67	0.698	80	0.297
> 62.5 years	131 (52)	63		76	
**Gender**					
Male	140 (55)	60	0.076	73	**0.019**
Female	113 (45)	71		84	
**UICC (TNM) stage**					
II	72 (28)	79	**0.010**	89	**0.010**
III	181 (72)	60		73	
**First tumour location**					
Colon	191 (76)	67	0.435	81	**0.050**
*Cecum*	*10 (5)*				
*Right*	*50 (26)*				
*Transverse*	*13 (7)*				
*Left*	*83 (44)*				
*Sigma*	*35 (18)*				
Rectum	62 (24)	61	0.522	68	
**Adjuvant Chemotherapy**					
FL+OXA	143 (56)	66	0.512	78	0.629
FL	110 (44)	64		77	
***HLA-G* 3’UTR SNPs**					
**+2960 14 bp INDEL**					
Del/Del	78 (31)	54	**0.036**	70	0.231
Ins/Del	115 (45)	70		79	
Ins/Ins	60 (24)	70		84	
Dominant Model	175 (69)	70	**0.010**	81	0.095
Recessive Model	60 (24)	70	0.411	84	0.329
**+3003 T>C**					
T/T	201 (79)	66	0.725	77	0.996
T/C	47 (19)	60		79	
C/C	5 (2)	80		80	
Dominant Model	52 (21)	63	0.667	80	0.935
Recessive Model	5 (2)	80	0.600	80	0.985
**+3010 C>G**					
C/C	94 (37)	68	0.162	80	0.213
C/G	119 (47)	67		80	
G/G	40 (16)	54		67	
Dominant Model	159 (63)	64	0.318	77	0.577
Recessive Model	40 (16)	54	0.063	67	0.079
**+3027 C>A**					
C/C	225 (89)	63	0.176	78	0.684
C/A	27 (11)	81		80	
**+3035 C>T**					
C/C	205 (81)	63	**0.051**	77	0.367
C/T	47 (19)	77		81	
**+3142 G>C**					
G/G	93 (37)	68	0.113	79	0.226
G/C	119 (47)	68		80	
C/C	41 (16)	52		67	
Dominant Model	160 (63)	64	0.366	77	0.627
Recessive Model	41 (16)	52	**0.038**	67	0.085
**+3187 A>G**					
A/A	145 (57)	68	**0.019**	82	**0.036**
A/G	93 (37)	65		76	
G/G	15 (6)	33		51	
Dominant Model	108 (43)	61	0.130	73	0.063
Recessive Model	15 (6)	33	**0.007**	51	**0.025**
**+3196 C>G**					
C/C	106 (42)	60	0.062	72	0.191
C/G	107 (42)	73		84	
G/G	40 (16)	59		79	
Dominant Model	147 (58)	69	0.074	82	0.076
Recessive Model	40 (16)	59	0.417	79	0.773

DFS, Disease Free Survival; OS, Overall Survival; SNPs, Single nucleotide polymorphisms; FL, Fluoropirymidine; OXA, Oxaliplatin; significant values (≤0.05) are shown in bold.

^1^ *P* values (log-rank test) given for the genotypes, the dominant and/or the recessive models.

**Fig 1:**
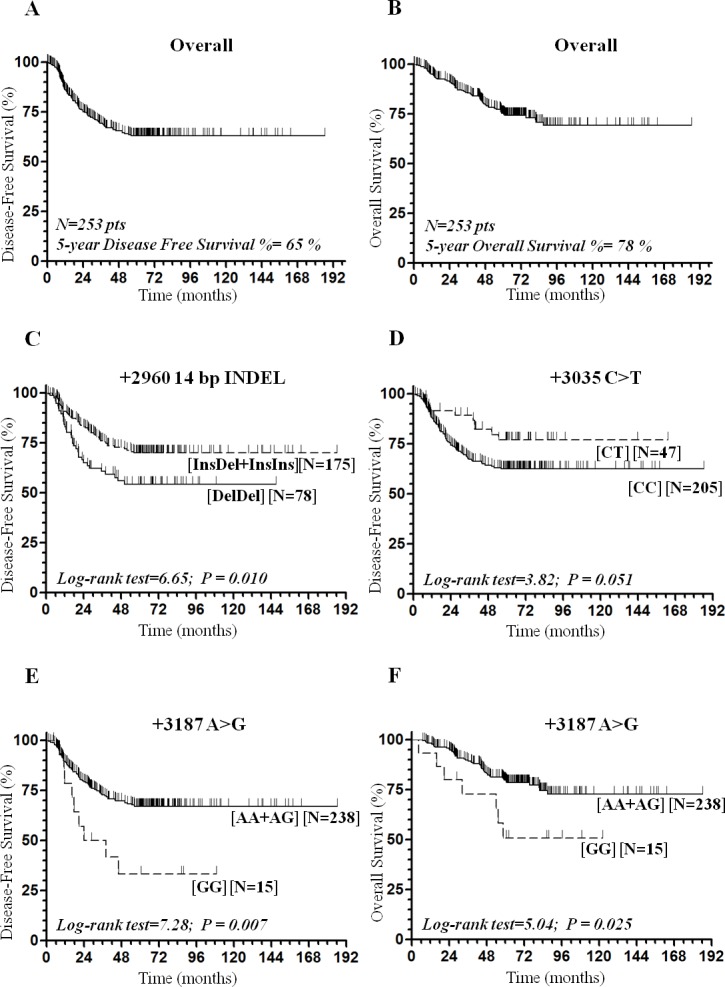
Kaplan-Meier survival curves for Disease Free Survival and Overall Survival according to the genotypes. **(A)** Disease Free Survival curve for the total of CRC patients. **(B)** Overall Survival curve for the total of CRC patients. **(C)** Disease Free Survival curve for the +2960 14-bp INDEL SNP according to the dominant model. **(D)** Disease Free Survival curve for the +3035 C>T SNP. **(E)** Disease Free Survival curve for the +3187 A>G SNP according to the recessive model. **(F)** Overall Survival curve for the +3187 A>G according to the recessive model.

Mean age was 60.5 +/-10.9 (IQ range 54–68 [25%-75%]) years, median age was 62.5 (range 24–82) years at onset. Most tumors (181/253 = 72%) presented stage III at the time of onset and were preferentially located at the left colon portion (191/253 = 76%). At diagnosis, stage II patients (N = 72) had pT3N0M0 (94%) of whom 6% had pT4aN0MO stage; most of them had >12 lymph nodes excised (72%), while 16% of patients had <12 lymph nodes excised. In 12% of cases the number of the analysed lymph nodes was not reported, although there was confirmation of no nodal involvement (N0). Mean follow-up time was 44.4 months for DFS (95% CI 42.1–46.7) and 74.8 months for OS (95% CI 71.9–77.8). Median follow-up time was 56.3 (range 1.2–186.3) months for DFS and 62.8 (range 4.6–186.3) months for OS. Five-year DFS was 65% (Fig 1A) and 5-year OS was 78% (Fig 1B). Total relapses were 82 (82/253 = 32%), 37% of staged III (67/181) and in 21% of stage II. Fifty six patients (56/253 = 22%) died during follow-up. Adjuvant therapy was administered as FL in 44% or as FL plus platinum (FL+OXA) in 56% of patients. One hundred and forty five patients (145/253 = 57%) completed the planned cycles for ADJ-CT. ADJ-CT treatment, FL alone or FL+OXA, was not significantly associated with both DFS (*P* = 0.512) and OS (*P* = 0.629) (Table 1). A significantly shorter OS was associated with males (*P* = 0.019), more advanced tumour stage (III) and rectal tumour location (*P* = 0.050) (Table 1). Moreover, advanced tumour stage was also associated with a shorter DFS (*P* = 0.010).

### *HLA-G* 3’UTR germinal screening

For each CRC patient the *HLA-G* 3’UTR segment was analyzed by direct sequencing. We detected 9 common SNPs at *HLA-G* 3’UTR: +2960 14-bp INDEL (rs371194629), +3003 T>C (rs1707), +3010 C>G (rs1710), +3027 C>A (rs17179101), +3035 C>T (rs17179108), +3142 G>C (rs1063320), +3187 A>G (rs9380142), +3196 C>G (rs1610696) and +3227 C>A (rs1233331). The germinal allele frequencies for *HLA-G* 3’UTR polymorphisms detected in this set of CRC patients were reported in S1 Table. Only those polymorphisms with ≥5% variant allelic frequency (+2960 14-bp INDEL, +3003 T>C, +3010 C>G, +3027 C>A, +3035 C>T, +3142 G>C, +3187 A>G, and +3196 C>G) were considered for survival (DFS and OS) analysis (Table 1). The distribution of genotypes of all selected SNPs were in agreement with HW equilibrium (S1 Table). Strong LD (r^2^ = 0.98) was found between +3010 C>G and +3142 G>C polymorphisms (S1 Fig). Finally, *HLA-G* 3’UTR haplotypes were reconstructed from the unphased gametic genotype data at the 16 variation sites for each of the 253 individuals affected by CRC by PHASE method. A total of 20 different haplotypes were defined; of these, 14 have been already described [46] and 7 (30%) were novel (for more details see S2 Table). The most represented haplotype was UTR-2 (36%), then UTR-1 (24%), UTR-3 (13%), UTR-4 (11%), UTR-7 (5%), UTR-5 (3%), UTR-18 (3%), UTR-15 (1%) and UTR-6 (1%). These 9 haplotypes represented more than 97% of total, but UTRs 1–4 were predominant (84%).

### Associations between *HLA-G* 3’UTR SNPs and Disease Free Survival

Among the 8 eligible polymorphisms identified, 4 SNPs (+2960 14-bp INDEL, +3035 C>T, +3142 G>C, and +3187 A>G) in the *HLA-G* 3’UTR region were associated to DFS in univariate analysis (Table 1). Statistically significant associations between DFS and the polymorphisms +2960 14-bp INDEL (log-rank *P* = 0.036), +3035 C>T (log-rank *P* = 0.051, Fig 1D), +3142 G>C in the recessive model (log-rank *P* = 0.038), and +3187 A>G (log-rank *P* = 0.019) were found (Table 1). The polymorphism +2960 14-bp INDEL was also associated to DFS in the dominant (Ins/Del + Ins/Ins *vs* Del/Del) model (log-rank *P* = 0.010, Fig 1C) and the +3187 A>G SNP in the corresponding recessive (G/G *vs* A/G+A/A) model (log-rank *P* = 0.007, Fig 1E). In particular, the Del/Del genotype was associated to a reduced 5-year DFS (54%) compared to both Ins/Del (70%) and Ins/Ins genotypes (70%) (Table 1). The +3035 C/T genotype was borderline significantly associated to prolonged 5-year DFS (77%) than the wild type +3035 C/C (63%) combination. Patients with the +3142 C/C mutated genotype had a reduced 5-year DFS (52% with respect to 68% in subjects carrying the wild type-G/G and heterozygous-G/C genotypes. The presence of the +3187 GG homozygous mutated genotype was associated with a reduced 5-year DFS (33%) compared to wild type +3187 A/A (68%) and the heterozygous +3187 A/G (65%) genotypes. UTR-1 haplotype, the second most represented (24%) in our CRC cohort, was associated to reduced DFS (log-rank *P* = 0.007, S2A Fig).

We further evaluated the association between the *HLA-G* 3’UTR SNPs and related reconstructed most abundant haplotypes (UTR2-, UTR-1, UTR-3, UTR-4, UTR-7, UTR-5, UTR-18, UTR-15 and UTR-6) and DFS of CRC patients by means of multivariate Cox models (Table 2 and Table 3 for haplotypes).

**Table 2:** Associations of *HLA-G* 3’UTR SNPs with disease free survival and overall survival in 253 stage II-III CRC patients.

	Disease Free Survival				Overall Survival		
*HLA-G* 3’UTR Genotypes and genetic models	N	Relapses, N	Adjusted HR (95% CI)^1^	*P-value*	Dead, N	Adjusted HR (95% CI)^1^	*P-value*
**+2960 14 bp INDEL** (rs371194629)							
Del/Del	78	33	1	-	22	1	-
Ins/Del	115	32	**0.59 (0.36–0.96)**	**0.035**	23	0.72 (0.40–1.30)	0.269
Ins/Ins	60	17	0.61 (0.34–1.10)	0.097	11	0.63 (0.30–1.30)	0.212
Dominant model *(Ins/Del+Ins/Ins)*	175	49	**0.60 (0.38–0.93)**	**0.023**	34	0.68 (0.40–1.18)	0.171
Recessive model (*Ins/Ins*)	60	17	0.82 (0.48–1.39)	0.457	11	0.76 (0.39–1.46)	0.406
**+3003 T>C** (rs1707)							
T/T	201	64	1	-	45	1	-
T/C	47	17	1.26 (0.73–2.17)	0.401	10	1.01 (0.51–2.03)	0.968
C/C	5	1	0.72 (0.10–5.22)	0.744	1	1.15 (0.16–8.42)	0.895
Dominant model *(T/C+C/C)*	52	18	1.21 (0.71–2.06)	0.480	11	1.03 (0.53–2.00)	0.942
Recessive model *(C/C)*	5	1	0.69 (0.10–4.96)	0.709	1	1.14 (0.16–8.36)	0.896
**+3010 C>G** (rs1710)							
C/C	94	27	1	-	19	1	-
C/G	119	38	1.11 (0.68–1.83)	0.670	24	0.99 (0.54–1.82)	0.973
G/G	40	17	1.63 (0.89–3.00)	0.115	13	1.60 (0.78–3.26)	0.197
Dominant model *(C/G+G/G)*	159	55	1.24 (0.78–1.96)	0.369	37	1.15 (0.66–2.00)	0.632
Recessive model *(G/G)*	40	17	1.54 (0.90–2.63)	0.117	13	1.61 (0.86–3.02)	0.140
**+3027 C>A** (rs17179101)							
C/C	225	77	1	-	50	1	-
C/A	27	5	0.54 (0.22–1.35)	0.186	5	0.82 (0.33–2.05)	0.666
**+3035 C>T** (rs17179108)							
C/C	205	72	1	-	47	1	-
C/T	47	10	**0.51 (0.26–0.99)**	**0.045**	8	0.66 (0.31–1.41)	0.287
**+3142 G>C** (rs1063320)							
G/G	93	27	1	-	19	1	-
G/C	119	37	1.05 (0.64–1.72)	0.860	24	0.96 (0.52–1.75)	0.882
C/C	41	18	1.65 (0.90–3.00)	0.103	13	1.54 (0.76–3.15)	0.233
Dominant model *(G/C+C/C)*	160	55	1.19 (0.75–1.89)	0.465	37	1.10 (0.63–1.93)	0.728
Recessive model *(C/C)*	41	18	1.61 (0.95–2.71)	0.078	13	1.59 (0.84–2.98)	0.153
**+3187 A>G** (rs9380142)							
A/A	145	42	1	-	26	1	-
A/G	93	31	1.16 (0.73–1.86)	0.529	23	1.26 (0.71–2.22)	0.431
G/G	15	9	**2.61 (1.24–5.50)**	**0.012**	7	**2.96 (1.22–7.15)**	**0.016**
Dominant model *(A/G+G/G)*	108	40	1.33 (0.86–2.05)	0.205	30	1.46 (0.86–2.48)	0.162
Recessive model *(G/G)*	15	9	**2.46 (1.19–5.05)**	**0.015**	7	**2.71 (1.16–6.33)**	**0.022**
**+3196 C>G** (rs1610696)							
C/C	106	40	1	-	29	1	-
C/G	107	26	0.61 (0.37–1.02)	0.059	18	0.68 (0.37–1.24)	0.206
G/G	40	16	0.96 (0.54–1.72)	0.899	9	0.72 (0.34–1.52)	0.388
Dominant model *(C/G+G/G)*	147	42	0.72 (0.46–1.12)	0.142	27	0.69 (0.41–1.18)	0.173
Recessive model *(G/G)*	40	16	1.18 (0.68–2.05)	0.559	9	0.84 (0.41–1.72)	0.630

^1^HRs (Hazard Ratios) adjusted for age, sex, tumour stage, first tumour site and type of adjuvant chemotherapy (FL plus or without OXA). Significant values (≤0.05) are shown in bold.

**Table 3:** Associations between *HLA-G* 3’UTR haplotypes with disease free survival and overall survival in 253 stage II-III CRC patients (N = 506).

		Disease Free Survival			Overall Survival		
*HLA-G* 3’UTR Haplotypes^1^	N	Relapses, N	Adjusted HR (95% CI)^2^	*P-value*	Dead, N	Adjusted HR (95% CI)^2^	*P-value*
**UTR-2**							
InsTCCCGA**G**G	180	42	1	-	26	1	-
Het	108	29	0.72 (0.44–1.18)	0.197	19	0.76 (0.43–1.37)	0.365
Hom	72	13	0.92 (0.49–1.73)	0.803	7	0.68 (0.29–1.55)	0.354
**UTR-1**							
DelT**G**CC**CG**CG	120	38	1	-	28	1	-
Het	90	29	1.08 (0.67–1.74)	0.745	21	1.12 (0.63–1.99)	0.699
Hom	30	9	**2.53 (1.20–5.32)**	**0.014**	7	**2.82 (1.17–6.77)**	**0.021**
**UTR-3**							
DelTCCCGACG	66	21	1	-	14	1	-
Het	56	19	1.09 (0.65–1.82)	0.753	13	1.11 (0.59–2.09)	0.745
Hom	10	2	1.98 (0.48–8.19)	0.347	1	1.15 (0.16–8.53)	0.890
**UTR-4**							
Del**CG**CC**C**ACG	55	17	1	-	11	1	-
Het	47	17	1.28 (0.74–2.19)	0.375	11	1.18 (0.61–2.31)	0.623
Hom	8	0	-	-	0	-	-
**UTR-7**							
InsTC**AT**GACG	27	4	1	-	5	1	-
Het	25	4	0.47 (0.17–1.29)	0.144	4	0.76 (0.27–2.13)	0.760
**UTR-5**							
InsTCC**T**GACG	17	4	1	-	3	1	-
Het	17	4	0.56 (0.21–1.54)	0.263	3	0.74 (0.23–2.39)	0.617
**UTR-18**							
DelT**G**CC**C**AC**A**	15	3	1	-	2	1	-
Het	15	3	0.55 (0.17–1.74)	0.307	2	0.57 (0.14–2.37)	0.441
**UTR-15**							
InsTCCCGACG	6	2	1	-	1	1	-
Het	4	2	1.91 (0.46–7.94)	0.371	1	0.87 (0.12–6.37)	0.893
**UTR-6**							
DelT**G**CC**C**ACG	6	3	1	-	1	1	-
Het	6	3	1.25 (0.38–4.16)	0.715	1	0.58 (0.08–4.33)	0.596

Het, haplotype in heterozygous state; Hom, haplotype in homozygous state; significant values (≤0.05) are shown in bold.

^1^ *HLA-G* 3’UTR haplotypes were reconstructed by PHASE method according to worldwide distributions^46^

^2^HRs (Hazard Ratios) adjusted for age, sex, tumour stage, first tumour site and type of adjuvant chemotherapy (FL plus or without OXA).

The Ins/Del heterozygous carriers had a significantly reduced risk or recurrence (HR 0.59, 95% CI 0.36–0.96, *P* = 0.035) as well *Ins* allele carriers of (low-HLA-G secretor) according to the dominant model (HR 0.60, 95% CI 0.38–0.93, *P* = 0.023). Forty-two percent of patients with the Del/Del genotype (N = 78) had a recurrence (33/78), while a lower incidence (28%) was observed in patients with Ins/Del (N = 115) and Ins/Ins (N = 60) genotypes (Table 2).

A borderline statistically significant association with reduced risk of disease recurrence was determined in patients carrying the +3035 C/T (low-HLA-G secretor) genotype (HR 0.51, 95% CI 0.26–0.99, *P* = 0.045). The 21% of heterozygous (C/T) patients for +3035 C>T SNP had a relapse (10/47) with respect to the 35% found in the wild type +3035 C/C combination (72/205). CRC patients carrying the +3187 G/G mutated genotype (high-HLA-G secretor) were associated with an increased risk or relapse (HR 2.61, 95% CI 1.24–5.50, *P* = 0.012 and consistently according to the recessive model (HR 2.46, 95% CI 1.19–5.05, *P* = 0.015). A detectable relapse was observed in the 60% (9/15) of CRC patients with the +3187 G/G mutated genotype (N = 15), in 33% (31/93) of heterozygous A/G (N = 93), and in 29% (42/145) of wild-type A/A (N = 145) patients.

UTR-1 haplotype, containing the +3187*G* mutated allele, was associated with poor prognosis in DFS when present in double (Hom) dose (HR 2.53, 95% CI 1.20–5.32, *P* = 0.014) (Table 3). The HR of the +3035 C/T and +2960 14-bp INDEL combined effect confirmed the protective role of both SNPs in DFS (HR 0.40, 95% CI 0.20–0.82, *P* = 0.013).

### Associations between *HLA-G* 3’UTR SNPs and Overall Survival

The +3187 A>G SNP was the only one to be associated with OS (log-rank *P* = 0.036), and in the corresponding recessive model (log-rank *P* = 0.025, Fig 1F) in univariate analysis (Table 1). Similarly, UTR-1 haplotype was associated to OS (log-rank *P* = 0.025, S2B Fig). In multivariate analysis carriers for the +3187 G/G genotype (high-HLA-G secretor) were associated with reduced OS (HR 2.96, 95% CI 1.22–7.15, *P* = 0.016) and consistently in the recessive model (HR 2.71, 95% CI 1.16–6.33, *P* = 0.022). Mortality rate was higher (47%) in homozygous mutated +3187 GG (7/15) carriers than in the heterozygous +3187 A/G (25%, 23/93) and in the wild type +3187 A/A (18%, 26/145) carriers (Table 2). Multivariate analysis for most represented haplotypes showed an association between UTR-1 haplotype in double (Hom) dose and diminished OS (HR 2.82, 95% CI 1.17–6.77, *P* = 0.021) (Table 3). The observed associations in both DFS and OS were unmodified in a bootstrap model confirming the internal validity of the associations observed (not shown).

The relation between types of polymorphisms and DFS or OS risk, was further examined stratifying by stage (II and III) (not shown). Although some differences in the hazard ratios were detected across strata, the observed associations were still confirmed. Moreover, these associations were compatible with the effect of random variation since heterogeneity tests were not significant.

## Discussion

Emerging data demonstrates a key role of genes involved in immune response checkpoints and their associations with the CRC clinical outcomes [47]. In order to progress, malignant tumours must elude or evade the host’s immune system. In the quest to develop personalized cancer therapies, researchers are increasingly examining the patient’s immune response to cancer. SNPs within genes involved in immune response should be helpful to define the immunogenetic profile of the patients and to improve treatment strategies modulating anti-tumor immune response by targeting novel immune checkpoints. Improvement in immunosurveillance mechanisms may be achieved by means of immunotherapies with monoclonal antibodies and through chemotherapies and radiotherapies [48]. The purpose of personalized medicine is to identify the optimal treatment for each individual patient to maximize benefits and minimize adverse effects. To achieve this goal, novel informative biomarkers and new approaches to optimize clinical outcomes are needed in order to better stratify patients for cancer care.

The potential clinical relevance of HLA-G in cancer as a negative regulator due to its direct or indirect tolerogenic properties to avoid immune cells response, was previously highlighted in several studies [17,24]. However, HLA-G molecule may counteract or elicit the progression of cancer as a consequence of its immune-modulatory properties regulated by SNPs present in the untranslated regions [15]. Previous studies have reported significant associations between *HLAG* polimorphisms (in particular the +2960 14-bp INDEL) and cancer risk [30,34], but to the best of our knowledge, this is the first study indicating a role for *HLA-G* 3’UTR regulatory SNPs in DFS and OS after adjuvant treatment of CRC. Our results emphasize the role of the host’s immunogenetic background in the CRC prognosis as well as report the molecular characterization of the 3’UTR region at the germinal level in subjects affected by colorectal cancer [42].

The common *HLA-G* 3’UTR polymorphisms were investigated and after multivariate survival analyses using Cox’s regression models we found that +2960 14-bp INDEL, +3035 C>T and +3187 A>G SNPs had a significant and independent prognostic role with high internal validity by bootstrap modeling. *HLAG* 3’UTR SNPs previously reported to be associated with a reduced protein production such as +2960 14-bp INDEL and +3035 C>T were linked to a better prognosis, whereas the +3187 A>G SNP (increased HLAG production), was associated to worse DFS and OS.

This study shows that the +2960 14-bp INDEL SNP, already described as a disease risk-marker, is also a prognostic marker for DFS in CRC patients treated with standard ADJ-CT. Intriguingly, our results on survival for +2960 14-bp INDEL SNP are in agreement with those observed in a non-solid tumour [31] and also in patients infected by the human immunodeficiency virus (HIV) [49], further highlighting the prognostic relevance for the 14-bp Ins/Del polymorphism. The +2960 14-bp INDEL (Ins/Del) SNP (rs371194629) was reported to modulate the magnitude of HLA-G production by regulating HLA-G mRNA stability [25]. In particular, the Del/Del genotype has been associated with high and stable *HLA-G* mRNA expression and higher levels of the soluble HLA-G, whereas the Ins/Ins genotype displays a lower production of mRNA and soluble or membrane bound molecules [29–32]. At the multivariate analysis, we estimated an association with a reduced DFS in patients who are carriers of *Ins* allele in the heterozygous Ins/Del patients and in (Ins/Del+Ins/Ins) agreement with the dominant model (HR 0.60, 95% 0.38–0.93, *P* = 0.023). CRC patients with the Del/Del genotype showed an increased relapse rate and reduced 5-year DFS %.

Concerning OS, we found a similar trend of improved prognosis for *Ins* allele even if not statistically significant probably due to the small sample size. In reference to SNP +3035 C>T (rs17179108), it has been reported, in a recent published study [29], that subjects presenting the +3035 C/T genotype had significantly lower levels of the soluble HLA-G compared to +3035 C/C (wild type) subjects. We observed a protective role of +3035 C/T genotype in the outcome (DFS) of CRC patients, though with a borderline (HR 0.51, 95% 0.26–0.99, *P* = 0.045) statistically significant association in the multivariate Cox’s model. The +3035 C/T genotype was associated to prolonged 5-year DFS (77%) and a lower (21%) recurrence incidence (10/47) with respect to the wild type +3035 C/C combination that presented a decreased 5-year DFS (63%) and a 35% of relapse (72/205). CRC carriers for +3035 C/C had a decreased 5-year DFS % and an increased relapse incidence. It should be pointed out that the +3035 C/T heterozygous genotype, detected in 47 patients, was always associated to the *Ins* allele, in heterozygous Ins/Del (N = 27) and in homozygous Ins/Ins (N = 20) patients. Even if a protective HR in DFS resulted in the combined analysis of +3035 C/T and +2960 14-bp INDEL polymorphisms, no firm conclusion about a multiplicative or additive effect of the 2 SNPs cannot be inferred from this study. Regarding the +3187 A>G polymorphism (rs9380142), the +3187*A* allele has been associated to decreased HLA-G expression and the presence of +3187 G/G genotype to significantly increased soluble levels of HLA-G [29,50]. To date, no association with survival and this *HLA-*G SNP was reported. We found an association between +3187 G/G carriers (HR 2.61, 95% CI 1.24–5.50, *P* = 0.012), and according to the recessive (G/G *vs* A/A+A/G) model (HR 2.46, 95% CI 1.19–5.05, *P* = 0.015) with reduced DFS. Similarly, the +3187 G/G carriers were associated to a reduced OS (HR 2.96, 95% CI 1.22–7.15, *P* = 0.016), also in the recessive model (HR 2.71, 95% CI 1.16–6.63, *P* = 0.022). These results may highlight that the modulation of the clinical outcome in CRC patients harbouring the +3187 A>G change is due to the contribution of the *G* allele in double dose. CRC patients carriers for +3187 G/G mutated genotype (N = 15) had increased recurrence rate and a reduced 5-year DFS %. Carriers of +3187 G/G mutated combination were also associated to a reduced 5-year OS percentage and increased mortality rate. The opposite prognostic associations found in CRC patients for SNPs +2960 14-bp INDEL and +3187 A>G, are corroborated by the evidence that these two polymorphisms are not in LD (r^2^ = 0.27). Furthermore, presence of +3187*G* allele is always associated to the *Del* allele, which are represented in the reconstructed UTR-1 haplotype. The latter, when present in double dose (UTR-1/UTR-1), has been associated to an unfavourable prognosis such as +3187 G/G genotype.

The variation sites described in the 3’UTR are mainly arranged in haplotypes (known as UTR-1 to UTR-44) with the UTR-1 and UTR-2 as the most frequent in the worldwide population [46]. Therefore, we performed a haplotype analysis on *HLA-G* 3’UTR variants to test whether haplotypes are more predictive than single variants.

UTR-1 (**Del**C**TGC**G**C**CGCGT**CGC**G) haplotype carrying the 14-bp *Del*, +3003*T*, +3010*G*, +3027*C*, +3035*C*, +3142*C*, +3187*G* and +3196*C* alleles, has been considered as a high expressing haplotype. In particular, individuals with the +3187 G/G genotype and thus the UTR-1/UTR-1 combination in double dose, exhibit significant higher levels of the soluble HLA-G [29,51]. UTR-1/UTR-1 haplotype combination shares characteristics of high HLA-G producer presenting the 14-bp Del/Del, the 3142 C/C and the 3187 G/G genotypes. UTR-1 was the only *HLA-G* 3’UTR haplotype associated with prognosis in CRC patients in multivariate regression Cox’s analysis. We found an association of reduced DFS (HR 2.53, 95% CI 1.20–5.32, *P* = 0.014) and OS (HR 2.82, 95% CI 1.17–6.77, *P* = 0.021) in CRC patients carrying the UTR-1 haplotype in double (Hom) dose. Estimations found are not surprising since patients homozygous for UTR-1 haplotype (N = 15) are the same carriers for +3187 G/G change and share the same survival pattern.

Moreover, we observed a strong LD (S1 Fig, available online) between the +3010 C>G (rs1710) and +3142 G>C (rs1063320) SNPs, consistently with the data reported for the worldwide population [46]. Both polymorphisms were not associated to DFS or OS in multivariate analysis (Table 2). In summary, our results demonstrates an independent potential prognostic value after multivariate analysis for three *HLA-G* 3’UTR polymorphisms, the +2960 14-bp INDEL, the +3035 C>T, and +3187 A>G. CRC patients carrying the *Ins* allele (lower HLA-G producer) were associated with a favourable DFS with a reduced risk of relapse (protective prognostic role). CRC patients carriers for the +3187 G/G genotype and UTR-1/UTR-1 haplotype (higher HLA-G producer) were associated with an unfavourable prognosis in both DFS and OS. Furthermore, patients carrying the +3035 C/T genotype (lower HLA-G producer) and therefore the *Ins* allele (in heterozygous or homozygous state) were associated with an improved prognosis though with a borderline significant association.

Finally, this study supports associations between the non-metastatic colorectal cancer outcome after CT treatment and polymorphisms of a gene involved in immune tumour escape. Our preliminary findings share a functional rationale considering that patients with higher levels of HLA-G would be more immunosuppressed and have a worse clinical prognosis as postulated by Rizzo *et al* [31]. The effect of immune surveillance on the outcome of patients after chemotherapeutic treatments is one of the currently attracting issues in cancer therapy. It is well recognized that the individual variability of drug response depends also on the genetic variations in human genome, thus enforcing the concept of personalized medicine [52]. Due to the functional impact of the HLA-G protein in cancer immune contexture, and the known correlations of functional-regulatory SNPs in the 3’UTR with the HLA-G protein level, the concept of germline genomic variation is very attractive. Only a small sample of blood is required for the genetic test, and the genotyping process is a standard and common method used in clinical practice. In addition, it should be especially considered for inoperable patients. Future perspectives will be analyze *HLA-G* 3’UTR polymorphisms in the outcome of metastatic CRC patients. These results could provide new insights to better stratify patients and also for combination therapy between CT and monoclonal antibodies (i.e. cetuximab and bevacizumab) currently adopted in clinical practice.

Some limitations of this work should be highlighted. Despite an internal validation (bootstrap analysis) confirming the results, *HLA-G* 3’UTR SNPs should be analyzed for their prognostic role in an independent CRC cohort treated with surgical resection and without any chemotherapy. Moreover, our data should be explored considering also the contribution of validated prognostic and predictive biomarkers for CRC. Another limit is the lack of biological samples such as tumour or plasma samples to correlate genetic data with tissue and/or soluble levels of HLA-G to sustain functional hypothesis about the regulatory role of these SNPs, even if previous published studies support consistent data [29–32,51]. Nonetheless, these novel findings presented here for the role of *HLA-G* 3’UTR region in prognosis of colorectal cancer provide the basis for implementation of personalized cancer treatments. Identification of high risk patients with well-recognized prognostic, predictive and novel immune-related genomic biomarkers may represent a new frontier in the management of CRC patients. In conclusion, +3035 C>T and in particular, +2960 14-bp INDEL and +3187 A>G polymorphisms in the regulatory 3’UTR of the *HLA-G* gene, have emerged as novel prognostic biomarkers in determining survival outcome in colorectal cancer. Therefore, our exploratory findings should be verified in independent larger CRC cohorts as well as further relevant functional studies are required.

## Supporting Information

S1 Fig Linkage Disequilibrium (LD) patterns at the 3’UTR region of *HLA-G* in 253 CRC patients. LD plot generated by Haploview shows correlations between all pairs of variants with MAF >2%. High pairwise LD (r^2^) between variants is illustrated with dark shading. The r^2^ values (x100) for the marker pairs are listed in the corresponding boxes.
(TIF)

S2 Fig Kaplan-Meier survival curves for Disease Free Survival and Overall Survival according to the UTR-1 haplotype. **(A)** Disease free survival curve for the UTR-1 haplotype in heterozygous (Het) and homozygous (Hom) CRC patients. **(B)** Overall survival curve for the UTR-1 haplotype in heterozygous (Het) and homozygous (Hom) CRC patients.
(TIF)

S1 Table Allele, genotype numbers and frequencies and HWE expectations observed at *HLA-G* 3’UTR polymorphic sites in 253 stage II-III CRC patients. (DOC)

S2 Table Haplotype numbers and frequencies observed at *HLA-G* 3’UTR polymorphic sites in 53 stage II-III CRC patients. (DOC)
