# Supplementary figures and images for: HLA-G 3’UTR Polymorphisms Impact the Prognosis of Stage II-III CRC Patients in Fluoropyrimidine-Based Treatment
Source: PLoS One. 2015 Dec 3;10(12):e0144000. doi: 10.1371/journal.pone.0144000 (PMC4669157; doi:10.1371/journal.pone.0144000)

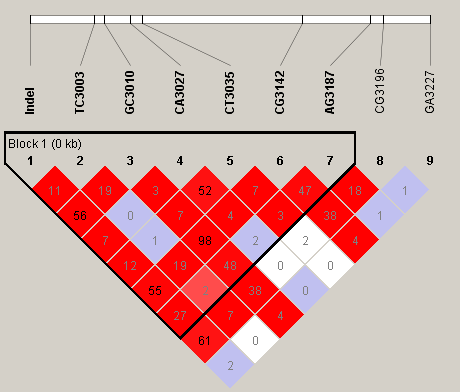

Supplement: S1 Fig — LD plot generated by Haploview shows correlations between all pairs of variants with MAF >2%. High pairwise LD (r2) between variants is illustrated with dark shading. The r2 values (x100) for the marker pairs are listed in the corresponding boxes. (TIF) [file pone.0144000.s001.TIF]

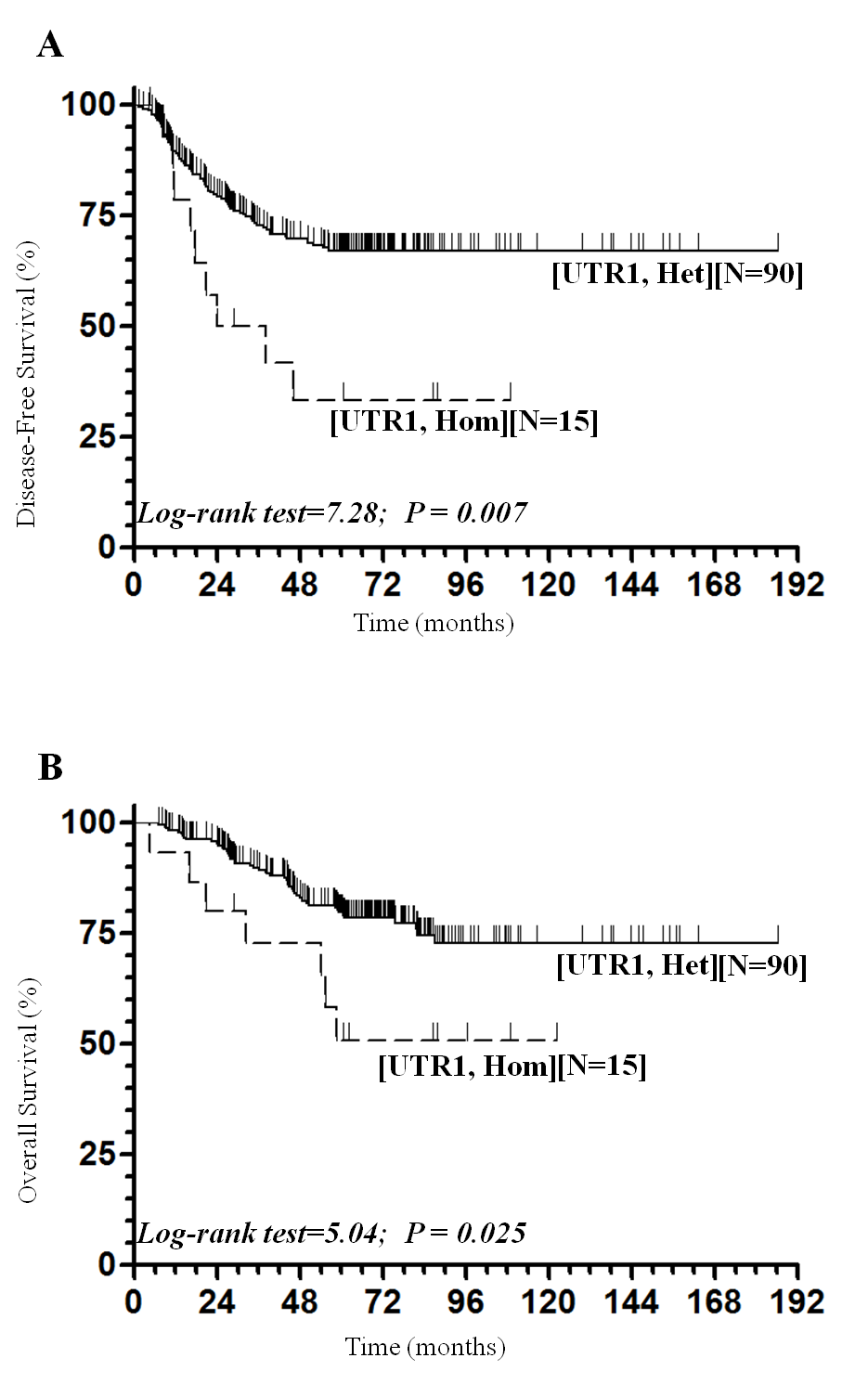

Supplement: S2 Fig — (A) Disease free survival curve for the UTR-1 haplotype in heterozygous (Het) and homozygous (Hom) CRC patients. (B) Overall survival curve for the UTR-1 haplotype in heterozygous (Het) and homozygous (Hom) CRC patients. (TIF) [file pone.0144000.s002.tif]
