# Supplementary material for: HLA-G 3’UTR Polymorphisms Impact the Prognosis of Stage II-III CRC Patients in Fluoropyrimidine-Based Treatment
Source: PLoS One. 2015 Dec 3;10(12):e0144000. doi: 10.1371/journal.pone.0144000 (PMC4669157; doi:10.1371/journal.pone.0144000)
Supplement: S1 Table — (DOC) [file pone.0144000.s003.doc]

**Supporting Table S1. Allele, genotype numbers and frequencies and HWE expectations observed at *HLA-G* 3’UTR polymorphic sites in 253 stage II-III CRC patients**

| ***HLA-G* 3’UTR SNPs in CRC** | **TOT, N=253** | **HWE^1^** |
| --- | --- | --- |
|  | **N (%)** | ***P*** |
| **+2960 14-bp INDEL** (rs371194629)  Del  Ins  Del/Del  Ins/Del  Ins/Ins | 271 (53.56)  235 (46.44)  78 (30.83)  115 (45.45)  60 (23.72) | 0.201 |
| **+3003 T>C** (rs1707)  T  C  T/T  T/C  C/C | 449 (88.74)  57 (11.26)  201 (79.45)  47 (18.58)  5 (1.97) | 0.385 |
| **+3010 C>G** (rs1710)  C  G  C/C  G/C  G/G | 307 (60.67)  199 (39.33)  94 (37.15)  119 (47.04)  40 (15.81) | 0.896 |
| **+3027 C>A** (rs17179101)  C  A  C/C  C/A  A/A | 477 (94.27)  29 (5.73)  225 (88.93)  27 (10.67)  1 (0.40) | 1.000 |
| **+3035 C>T** (rs17179108)  C  T  C/C  C/T  T/T | 457 (90.32)  49 (9.68)  205 (81.02)  47 (18.58)  1 (0.40) | 0.589 |
| **+3142 G>C** (rs1063320)  G  C  G/G  G/C  C/C | 305 (60.28)  201 (39.72)  93 (36.76)  119 (47.04)  41 (16.20) | 0.853 |
| **+3187 A>G** (rs9380142)  A  G  A/A  A/G  G/G | 383 (75.69)  123 (24.31)  145 (57.31)  93 (36.76)  15 (5.93) | 1.000 |
| **+3196 C>G** (rs1610696)  C  G  C/C  C/G  G/G | 319 (63.04)  187 (36.96)  106 (41.90)  107 (42.29)  40 (15.81) | 0.172 |
| **+3227 G>A** (rs1233331)  G  A  G/G  G/A  A/A | 491 (97.04)  15 (2.96)  238 (94.07)  15 (5.93)  0 | 1.000 |

SNPs, Single nucleotide polymorphisms; HWE, Hardy-Weinberg equilibrium; significant values (< 0.05) are shown in bold.

^1^probability of adherence to the HWE expectations was calculated only for SNPs with MAF >2%.
