# Supplementary material for: HLA-G 3’UTR Polymorphisms Impact the Prognosis of Stage II-III CRC Patients in Fluoropyrimidine-Based Treatment
Source: PLoS One. 2015 Dec 3;10(12):e0144000. doi: 10.1371/journal.pone.0144000 (PMC4669157; doi:10.1371/journal.pone.0144000)
Supplement: S2 Table — (DOC) [file pone.0144000.s004.doc]

**Supporting Table S2. Haplotype numbers and frequencies observed at *HLA-G* 3’UTR polymorphic sites in 253 stage II-III CRC patients**

| ***HLA-G* 3’UTR Haplotypes in CRC^1^** | **CRC**  **(N=506)** | **Frequency** |
| --- | --- | --- |
| **UTR-1**  **Del**CT**G**CG**C**CGCGT**CG**CG | 120 | 0.237 |
| **UTR-2**  **Ins**CTCCG**C**CGCGTGA**G**G | 180 | 0.356 |
| **UTR-3**  **Del**CTCCG**C**CGCGTGACG | 66 | 0.130 |
| **UTR-4**  **Del**C**CG**CG**C**CGCGTCACG | 55 | 0.109 |
| **UTR-5**  **Ins**CTCCGTCGCGTGACG | 17 | 0.034 |
| **UTR-6**  **Del**CT**G**CG**C**CGCGT**C**ACG | 6 | 0.0120 |
| **UTR-7**  **Ins**CTC**A**GTCGCGTGACG | 27 | 0.053 |
| **UTR-10**  **Del**CTCCG**C**CGCGTGA**G**G | 2 | 0.004 |
| **UTR-15**  **Ins**CTCCG**C**CGCGTGACG | 6 | 0.0120 |
| **UTR-18**  **Del**CT**G**CG**C**CGCGT**C**AC**A** | 15 | 0.030 |
| **UTR-20**  **Del**CT**G**C**CC**CGCGT**C**ACG | 1 | 0.002 |
| **UTR-38**  **Del**CTCCG**C**CGCGTG**G**CG | 1 | 0.002 |
| **UTR-44**  **Ins**CTCCGTCGCG**C**GACG | 1 | 0.002 |
| **UTR-New1**  **Del**CTCCGTCGCGTGAGG | 2 | 0.004 |
| **UTR-New2**  **Del**CTCCGCCGCGTCA**C**G | 1 | 0.002 |
| **UTR-New3**  **Del**CCGCGCCGCGTCGCG | 2 | 0.004 |
| **UTR-New4**  **Ins**CTCAGCCGCGTGAGG | 1 | 0.002 |
| **UTR-New5**  **Ins**CTCAGTCGCGTGAGG | 1 | 0.002 |
| **UTR-New6**  **Ins**CTCCGTCGCGTCACG | 1 | 0.002 |
| **UTR-New7**  **Ins**CTCCGCCTCGTGAGG | 1 | 0.002 |

^1^*HLA-G* 3’UTR haplotypes were reconstructed by PHASE method according to worldwide distributions [46].
